# Hemispheric Processing of Chinese Scientific Metaphors: Evidence *via* Hemifield Presentation

**DOI:** 10.3389/fpsyg.2022.894715

**Published:** 2022-05-26

**Authors:** Min Huang, Lexian Shen, Shuyuan Xu, Yanhong Huang, Shaojuan Huang, Xuemei Tang

**Affiliations:** ^1^School of Foreign Studies, Anhui Polytechnic University, Wuhu, China; ^2^Key Laboratory of Modern Teaching Technology, Ministry of Education, Shaanxi Normal University, Xi’an, China

**Keywords:** scientific metaphor, divided visual field diagram, hemispheric asymmetry, N400, LPC

## Abstract

The role of the two hemispheres in processing metaphoric language is controversial. In order to complement current debates, the current divided visual field (DVF) study introduced scientific metaphors as novel metaphors, presenting orientation mapping from the specific and familiar domains to the abstract and unfamiliar domains, to examine hemispheric asymmetry in metaphoric processing. Twenty-four Chinese native speakers from science disciplines took part in the experiment. The participants were presented with four types of Chinese word pairs: scientific metaphors, conventional metaphors, literal word pairs, and unrelated word pairs. The first word in each pair was presented centrally, and the second was presented to the left visual field (the Right Hemisphere) or the right visual field (the Left Hemisphere). Event-related potentials (ERPs) were recorded when participants read the target words and judged whether words in each pair were related. The data demonstrated that both hemispheres were involved at the initial stage of metaphor processing, but the right hemisphere took a more privileged role. The significant activation of the left hemisphere for scientific metaphoric processing supports the fine-coarse coding hypothesis. During right-visual-field presentation, the left hemisphere, responsible for the processing of closely related domains, has to integrate the loosely associated domains of scientific metaphor, which greatly increased cognitive taxes. Moreover, the data of late positive components (LPCs) revealed different hemispheric activation between scientific metaphors and conventional metaphors. Compared with literal pairs, conventional metaphors elicited significantly higher LPCs during right visual field presentation, while the scientific metaphor elicited significantly lower LPCs during left visual field presentation. These results suggest different processing mechanisms between novel metaphors and conventional metaphors and the special role of the right hemisphere in novel metaphoric processing at the later mapping stage.

## Introduction

Metaphors are used pervasively in our daily life to enable more effective and efficient communication. As in “*Marriage is gambling*,” two conceptual areas are equated, and readers can easily go beyond the literal meaning to grasp the figurative meaning by analogizing or comparing the two concepts. In previous research works, metaphors were categorized into conventional metaphors and novel metaphors according to their familiarity or conventionality, determining those which can be comprehended effortlessly by the native speakers as conventional metaphors. To date, much attention has been paid to the hemispheric processing of metaphors. However, there is still no agreement, especially on the role of the right hemisphere in novel metaphoric processing.

To complement the current debate, we attempted to further differentiate novel metaphors. The scientific metaphors we used in this study are rather imaginative and often used by scientists to reason or communicate abstract scientific concepts. For example, through the scientific metaphor, “*sound wave*,” we may understand the characteristics of “*sound*” comparatively more easily through the concept of “*wave*.” Similar to the novel metaphors used in most previous research, the scientific metaphors we used in our study were non-conventional and unfamiliar. However, they have a “more complicated contextual structure” involving a longer mapping distance (Tang et al., 2017a). The source domain of wave (as in “*water wave*”) is derived from daily-use contexts, while the target domain in which the metaphor is applied as “*sound*” is from scientific contexts, which increases the difficulty of integration. Besides, the late processing of scientific metaphors involves a unique reasoning process to understand the related knowledge, which taxes semantic reintegration. Using scientific metaphors to study hemispheric processing might shed some light on relevant studies.

### Lateralization in Metaphor Processing

Most neuro-linguists and psychologists agree that metaphorical meaning processing is different from literal meaning processing with the former showing a preference for the right hemisphere. Compared with the theory of the left hemisphere involvement in metaphorical processing, *the right hemisphere engagement theory* does gain more attraction, because it fits into a more holistic picture of the brain’s division as described in Jung-Beeman’s (2005) fine-coarse hypothesis: the left hemisphere is primarily responsible for the fine coding of closely related meanings, while the right hemisphere is for the coarse coding of non-literal meaning, including metaphors.

Moreover, some clinical studies involving schizophrenia, Asperger’s syndrome, Alzheimer’s disease, and other pathologies have reported irregular lateralization of metaphor processing, indicating the important role of the right hemisphere in metaphor processing (Rapp et al., 2004; Lauro et al., 2008; Ianni et al., 2014). In addition, some experimental studies on healthy subjects also confirmed the right-hemisphere advantage in metaphor processing. For example, a positron emission tomography study found that the processing of metaphorical sentences resulted in increased blood flow in the prefrontal cortex, middle temporal gyrus, anterior cingulate gyrus, and posterior cingulate gyrus of the right hemisphere, compared with the processing of the literal meaning of the same structure (Bohrn et al., 2012). Some fMRI (Yang, 2014; Lai et al., 2015) and Event-related potential (ERP) studies (Tang et al., 2017a,b) also suggested that the right brain played an important role in metaphor processing.

Different linguists have given different explanations for the special role of the right hemisphere in metaphor processing. According to the Fine Coarse Semantic Coding Model (Beeman, 1998, 2005), the right hemisphere has an advantage in semantic processing with a large semantic span, while the left hemisphere is mainly responsible for processing conventional concepts of words. However, based on the graded salience hypothesis (GSH) (Giora, 1997, 2003), the right hemisphere has an advantage in low salient meaning processing, while the left hemisphere has an advantage in high prominent meaning processing (Mashal and Faust, 2008).

In contrast to these findings, other studies reported no predominance of the right hemisphere during non-literal language processing. Some experiments have found that the left hemisphere advantage was found in figurative language comprehension (Eviatar and Just, 2006; Bohrn et al., 2012; Mitchell et al., 2016), while other studies reported that both hemispheres are involved in metaphoric meaning processing (Yang et al., 2016).

Previous studies have found that the salience, conventionality, familiarity, anticipation, predictability, or transparency of metaphors and other figurative languages used in those experiments can modulate the results produced to a large extent. For example, a study on idioms argued that the predictability of metaphors was the main factor that decided the means of semantic processing (Sela et al., 2017). In addition, according to *the metaphor career theory* (Bowdle and Gentner, 2005), metaphors with different degrees of conventionality have different neural mechanisms for processing. According to *the structure mapping theory* (Wolff and Gentner, 2011), metaphor processing involves an initial processing stage of symmetric alignment and a later directional phase in which inferences are projected to the target. More importantly, Wolff and Gentner (2011) proposed that the base of highly conventional metaphors already possesses a salient conventional metaphoric meaning, whereas metaphoric abstraction must be derived anew for a novel figurative. However, some early experiments only distinguished literal meaning from metaphorical meaning without a further classification of metaphors (Eviatar and Just, 2006; Bohrn et al., 2012; Mitchell et al., 2016). Most current studies differentiated conventional metaphors from novel metaphors without further classifying novel metaphors. One of our previous studies adopted a central visual presentation paradigm to compare Chinese poetic and scientific metaphors in sentence context and reported larger late negativity in the LPC window simultaneously on the left and the right hemispheres suggesting both hemispheres of the brain work together when processing scientific metaphors (Tang et al., 2017b). There is an urgent need to study some specific types of novel metaphors to observe the lateralization in metaphor processing.

### Hemifield Priming and Metaphor Processing

Many previous research works adopted a divided visual field (DVF) priming paradigm to study the hemispheric effect of metaphor processing. Presenting stimuli in the right or left visual field can control information selectively activating only the left or right visual cortex during the initial stage of language processing. In other words, for normal individuals, information is rapidly transmitted to the opposite hemisphere.

Meanwhile, in traditional behavioral DVF experiments, researchers often modulated stimulus onset asynchrony (SOA) to study lateralization as well as the dynamic time course during metaphor comprehension. Anaki et al. (1998) found that when SOA was short (200 ms), both literal and metaphorical pairs produced a left-brain priming effect, but only metaphorical words produced a right-brain priming effect. When the SOA was long enough (800 ms), only literal words had left-brain priming effects, while metaphorical words had right-brain priming effects. These findings suggested that metaphorical meaning is initially activated in both hemispheres, but the degree of activation declines rapidly in the left hemisphere but remains constant in the right hemisphere. Anaki’s experiment did not distinguish the semantic salience of the metaphorical corpus. Faust and Mashal (2007) divided the metaphor sentences into novel metaphors and conventional metaphors in their study. The study found that, under the conditions of SOA of 400 and 1,100 ms, both literal meaning and metaphorical meaning showed priming effects in the left and the right hemispheres, which indicated that both hemispheres worked together during metaphor comprehension in different stages. However, novel metaphors were processed faster and more accurately in the right hemisphere, supporting the right hemisphere theory. The rationale they put forward was that the left brain used sentence constraints to select and integrate literal and metaphorical meanings related to the context of the sentence. The right brain might be less sensitive to sentence context and only participate in alternative interpretations in cases where the literal meaning cannot be explained. However, using the same DVF paradigm, Forgács et al. (2014) found that novel metaphor pairs, like literal and conventional word pairs, have no significant right hemisphere effect and are processed faster and more accurately in the left hemisphere.

In addition, the DVF paradigm was sometimes combined with human electrophysiology to study non-literal language processing. A study reported that jokes presented to the right visual field-left hemisphere (rvf-LH) elicited a larger N400 than the non-joke endings; however, when presented to the left visual field-right hemisphere (lvf-RH), the joke and non-joke endings elicited N400s of equal amplitude (Coulson et al., 2005), supporting the idea that the right hemisphere plays a special role. Another hemifield study on metaphor processing, on the contrary, found that ERP metaphoricity effects were very similar across hemifields, suggesting that the integration of metaphoric meanings was similarly taxing for the two hemispheres (Coulson and Van Petten, 2007). A DVF fMRI study on Chinese idioms reported similar results (Yang et al., 2016).

### The Current Study

In order to address the current debates on lateralization during metaphor processing, the present study chose Chinese scientific metaphors to assess the hemispheric processing of novel metaphors *via* a combination of the DVF paradigm and ERP methodology. Both source and target domains of conventional metaphors come from daily life, presenting symmetrically analogical mapping in which a structural alignment between two represented situations is established. For example, “*月亮 (crescent moon)*” and “*小船 (boat)*” have similar shapes and their associated abstract schemata are sufficiently accessible. However, source domains in scientific metaphors are abstract scientific concepts, the processing of which often needs to activate specific concepts in target domains. When understanding the term “*电流 (electric current)*,” it is hard to consider it as a pure category name. It involves a comparison between “*电子 (electronics)*” and “*水流 (current)*,” presenting orientation mapping from the specific and familiar domains to the abstract and unfamiliar domains. We compare “*human eyes*” to “*cameras*” as they work in a similar way.

When considering the notions of a “*sound wave*” or an “*electric current*,” the everyday concepts “*wave*” and “*current*” can help readers quickly understand certain scientific concepts “*sound*” or “*electricity*.” Such mapping across daily and scientific concepts requires semantic integration based on the adequate comparison, which results in increased cognitive load in retrieving stored conceptual knowledge and in integrating seemingly unrelated information from different domains in the process of metaphoric comprehension. Our previous research also proved that scientific metaphors elicited a diverse LPC from novel poetic metaphors and conventional metaphors (Tang et al., 2017b).

Despite limitations on spatial resolution, ERP technology has excellent performance on temporal revolution. Compared with other methodology, ERPs provide a continuous measure of word processing that is sensitive to lateralized brain activity over the different stages of processing. The combination of ERP and DVF techniques can help study lateralization during metaphor processing in a more accurate and dynamic way.

Our study replicated Coulson and Van Petten’s (2007) research, but used different stimuli and contexts. We used word pairs instead of sentences to avoid the influence of sentence contexts. Moreover, we replaced the novel metaphors with scientific metaphors as their source domain and target domain are from different contexts and therefore have a longer mapping distance. According to structure mapping theory, we assumed that scientific metaphors would elicit more negative N400s or other late negativity components. More importantly, we supposed that the presentation side of stimuli would interact with the ERP effects of metaphoric variables. If hemispheric differences in semantic activation affect metaphor comprehension, the presentation side (visual field) would be expected either to facilitate processing or to make it more difficult.

In the previous ERP studies, the amplitudes of N400 were reported to indicate the degree of difficulties in retrieving and integrating contextual meaning in metaphor comprehension (Lai and Curran, 2013; Schneider et al., 2014). Metaphors should thereby elicit a more negative N400 than literal meaning. In addition, LPC as a late component was reported to reflect a secondary integration of meaning required by novel metaphors (Kazmerski et al., 2003; Rutter et al., 2012; Tang et al., 2017a,b). Therefore, if the right hemisphere takes a special role in metaphor comprehension, our hypotheses are as follows.

(1) The amplitude of N400s elicited by scientific metaphors should be larger than those of conventional metaphors and literal pairs with the presentation to both hemifields.
(2) There should be some interaction between conditions and sides. Specifically, the differences between N400s and LPCs elicited by scientific metaphors, conventional metaphors, and literal pairs should be more significant with the presentation to rvf-LH than lvf-RH.

## Experimental Procedure

### Participants

Twenty-four undergraduate students (14 males, 10 females, average age 21.5) from Anhui Polytechnic University, aged 18–22, participated in the ERP experiment. All the participants were from science disciplines considering the possible difficulties in understanding the academic knowledge involved in scientific metaphors. All were native Chinese speakers and yielded a laterality quotient of at least +80 on the Edinburgh Inventory, indicating right-handedness (Oldfield, 1971). Exclusion criteria were sinistrality, past or present psychiatric illness or neurological disorder, or major head injury. All participants had normal or corrected to normal vision and were given monetary compensation for their participation. The experimental standards of the study were approved by the local Review Board for Human Participant Research. Each subject provided written informed consent before participating. They were presented with four types of word pairs and asked to perform semantic judgment on the second word of each pair that was presented to the rvf-LH or lvf-RH. Four participants had to be excluded from data analyses due to low accuracy in semantic judgment tasks, resulting in a final sample size of 20 subjects (12 males, 8 females, average age of 21.55).

### Stimuli

The stimuli of the experiment were better calibrated based on the corpus of ERP experimental studies on the neural mechanism of Chinese metaphor understanding (Tang et al., 2017a). The stimulus pool consisted of 375 pairs of words, all in Chinese. They were grouped into four types of semantic relations: literal pairs (LT: *debris flow 泥石流*, *disaster 灾害*), conventional metaphoric (CM: *eyes 眼睛*, *window 窗口*), scientific metaphoric (SM: *electric current 电流*, *current 水流*), or unrelated pairs (UR: *balcony 阳台*, *Antarctic Circle 南极圈*) (see Table 1 for examples).

**TABLE 1:** Chinese sample stimuli.

	Word 1	Word 2	English meaning of Word 1	English meaning of Word 2
Scientific metaphors	淋巴	警察	Lymph	Police
SM	电子	行星	Electrons	Planets
	导体	隧道	Conductors	Tunnels
	染色体	姐妹	Chromosomes	Sisters
	病毒	杀手	Virus	Killer
Conventional	语言	桥梁	Language	Bridge
metaphors	杭州	天堂	Hangzhou	Heaven
CM	家庭	港湾	Home	Harbor
	手机	伙伴	Cellphone	Partner
	恋爱	咖啡	Love	Coffee
Literal expressions	教授	学者	Professor	Scholar
LT	汉语	语言	Chinese	Language
	北京	城市	London	City
	蚂蚁	昆虫	Ant	Insect
	小狗	宠物	Dog	Pet

The first word of each pair served as the prime and the second as the target. Each word contained two or three Chinese characters. Besides, there were a certain number of scientific words in both literal and unrelated word pairs and all the scientific terms or concepts were collected from middle school or high school textbooks, so as to achieve a balance between different conditions in terms of word frequency.

Prior to the neurophysiological study, several pretests were conducted by raters who did not participate in the ERP experiment. Firstly, to determine the degree of semantic relatedness for the word pairs in each condition, 60 raters were presented with a list containing all 375-word pairs (75 scientific metaphoric word pairs, 75 conventional, metaphoric word pairs, 75 literal word pairs, and 150 unrelated word pairs) and asked to determine the plausibility and familiarity of all the word pair on a 1–5 scale (1 = not plausible/familiar, 5 = extremely congruent/familiar). Then another 60 raters were asked to decide the figurativeness of the scientific metaphoric, conventional metaphoric, and literal word pairs on a 1–5 scale (1 = not figurative, 5 = extremely figurative).

According to the results of the plausibility and familiarity judgments (see Table 2 for the descriptive data of pretests), 40 pairs with familiarity and plausibility over 3.5 (average rating of 4.1) and figurativeness below 1.7 (average rating of 1.4) were selected as the literal pairs, and 60 pairs with familiarity and plausibility below 1.7 (average rating of 1.4) were selected as the filler pairs. Among the 75 pairs of scientific metaphors, pairs with figurativeness lower than 2.5 were removed, and the remaining pairs were selected again according to the degree of familiarity and plausibility. Finally, 40 pairs with an average familiarity of over 2.9, plausibility over 3.5, and figurativeness over 3.3 were chosen as scientific metaphors. In 75 pairs of daily metaphors, pairs with figurativeness lower than 3 were eliminated, and then the remaining pairs were further evaluated according to their plausibility and familiarity. Finally, 40 pairs with an average figurativeness (see Table 2), plausibility and familiarity rating of 3.8, 3.8, and 3.6 were selected as daily metaphorical word pairs respectively. A repeated-measures ANOVA yielded significant differences (*p*s < 0.01) between word pair types in all dimensions.

**TABLE 2:** The results of pretests.

	Meaningfulness		Figurativeness		Familiarity	
	*M*	SD	*M*	SD	*M*	SD
SM	3.5	0.6	3.3	0.24	2.85	0.59
CM	3.8	0.42	3.79	0.24	3.64	0.45
LT	4.09	0.44	1.4	0.19	4.12	0.24
UR	1.45	0.2			1.43	16.24

### Procedures

The experiment took place in a sound-attenuated, electrically shielded room. Before the experiment, considering the possible difficulties in understanding the academic knowledge related to scientific words, we let participants read a list of the relevant terms alongside their definitions. Participants also had the opportunity to look up any unfamiliar ones using their cell phones or consult us to verify meanings. Participants were required to put their jaws on a stent fixed on a small table. The distance between the eyes of the participants and the display screen was 60 cm. The participants were asked to judge whether the priming words (*电荷/electronics*) and the target words (*水流/current*) were semantically related. Semantic relevance was defined as some similarities in appearance, nature, function, and working principle between priming words and target words, and irrelevance was defined as no similarities. E-Prime 2.2 was used to edit and present stimuli. Four types of word pairs were presented pseudo-randomly. Each word was presented in white on a black background. In terms of the font used, we opted for a regular script at a font size of 50. The first word of each pair was presented in the central field of vision as a priming word, and then a target word was presented in the left or right field of vision, with an average angle of 1.9° to the fixation in the central field. ERPs were recorded when participants read the target words and indicated whether words in each pair were semantically related. Behavioral data of semantic judgment was also recorded.

**FIGURE 1:**
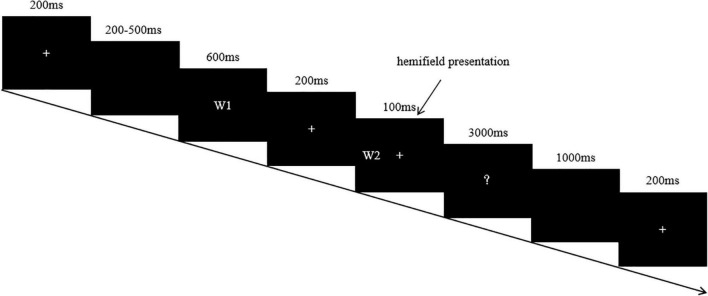
Experimental paradigm. (W1 and W2 refer to word 1 and word 2 respectively).

Stimuli on each trial were presented in the following time sequences: fixation cross (200 ms), blank (200–500 ms), priming word (600 ms), fixation cross (200 ms), target word (100 ms), and question mark (3,000 ms). Our reason for selecting an 800 ms SOA was that the metaphoric meaning is more adequately processed under this condition according to previous research. At the sight of the question mark, participants gave their judgment on whether the two words in each pair were semantically related or not. This was done by pressing a corresponding key as quickly/accurately as possible, using their left or right index fingers. An upper time limit of 3,000 ms was permitted for responses and was followed by a 1,000 ms inter-trial interval. The overall sequence of events for a trial is illustrated in Figure 1. Before the main session of the experiment, there was a brief practice session to familiarize the participants with the experimental procedure.

### EEG Recording and Analysis

EEG readings were continuously recorded from 64 scalp sites at a sampling rate of 512 Hz. Electrode impedance was kept below 5 kΩ. EEG epochs were synchronized with the onset of stimulus presentation and analyzed by all-caps MATLAB. Computerized artifact rejection was performed before averaging to discard epochs in which eye movements, blinks, excessive muscle potentials, or amplifier blocking occurred. EEG epochs associated with an incorrect behavioral response were also excluded. The artifact rejection criterion was a peak-to-peak amplitude exceeding 50 μV. This resulted in a rejection rate of ∼5%. ERPs were averaged off-line from −200 ms before stimulus onset to 1,000 ms after. ERP components were identified and measured, with reference to the average baseline voltage over the interval from −100 to 0 ms, at sites and latency where they reached their maximum amplitude.

## Results

### Behavioral Results

**FIGURE 2:**
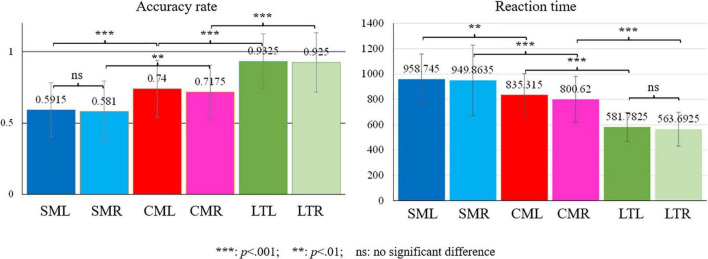
Behavioral results. (The third letter after stimulus type refers to the visual field. For example, SML refers to scientific metaphors presented to left visual field).

A 3 condition (literal, conventional, scientific) × 2 view (lvf, rvf) two-way ANOVAs yielded significant main effects of condition for reaction time [*F*(2,38) = 62.95, *p* = 0.000, η*_*p*_*^2^ = 0.768]. Pairwise comparison showed that the reaction time of scientific metaphors was significantly longer than that of conventional metaphors and literal pairs (*p*s = 0.000), while the reaction time of conventional metaphors was also significantly longer than that of literal pairs (*p* = 0.000). For accuracy rate, a main effect between conditions was found [*F*(2,38) = 39.985, *p* = 0.000, η*_*p*_*^2^ = 0.678]. Pairwise comparison showed that the accuracy rate of scientific metaphors was significantly lower than that of conventional metaphors and literal pairs (*p*s = 0.000), while the accuracy rate of conventional metaphors was significantly lower than that of literal pairs (*p* = 0.000). No distinguished hemifield presentation effect, as well as interactions with the condition, was found for reaction time and accuracy rate. Statistical results were shown in Figure 2.

### Electrophysiological Results

According to the previous research, we chose Fz, Cz, CPz, Pz as midline, F3, FC3, C3, CP3, P3, F4, FC4, C4, CP4, P4 as dorsal area, F7, FT7, T7, TP7, P7, F8, FT8, T8, TP8, P8 as ventral area, and F7, F3, Fz, F4, F8, FT7, FC3, FC4, FT8 as an anterior area, T7, C3, Cz, C4, T8 as a central line, and TP7, CP3, CPz, CP4, TP8, P7, P3, Pz, P2, P4, P8 as a posterior area in order to further analyze the hemisphere processing in metaphors (as shown in Table 3).

**TABLE 3:** Electrodes chosen for data analysis.

	Left	Midline	Right
Frontal	F7, F3, FT7, CP3	Fz	F4, F8, FT4, FC8
Central	T7, C3	Cz	C4, T8
Parietal	TP7, CP3, P7, P3	CPz, Pz	TP4, CP8, P4, P8

The resulting amplitudes of N400 and LPC were entered into 3 condition × 2 view (rvf, lvf) × 3 region (frontal, central, parietal) × 3 brain area (left, midline, right) four-way ANOVAs for repeated measures.

### 100–200 ms

**FIGURE 3:**
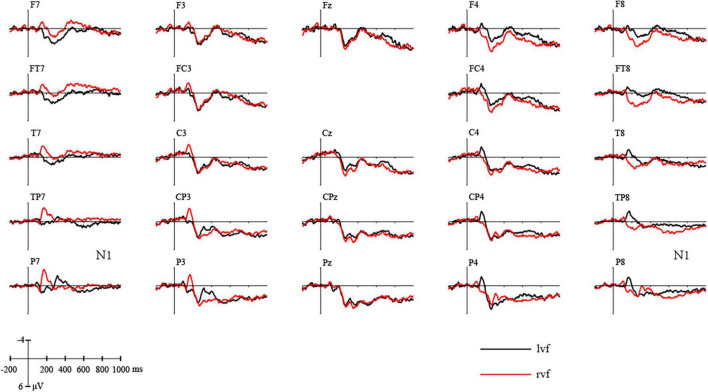
Difference curves of the grand average ERPs elicited by stimuli presented on left visual field and right visual field.

In the time window of N1 (100–200 ms), consistent with Figure 3, a repeated-measures ANOVA showed a significant view × hemisphere interaction [*F*(2,38) = 25.337, *p* = 0.000, η*_*p*_*^2^ = 0.571]. *Post hoc* analysis showed that in the left hemisphere, stimuli presented to rvf-LH elicited significantly higher N1 than stimuli presented to lvf-RH [*F*(1,19) = 12.674, *p* = 0.002, η*_*p*_*^2^ = 0.400], while in the right hemisphere, stimuli presented to lvf-RH elicited significantly higher N1 than stimuli presented to rvf-LH [*F*(1,19) = 15.433, *p* = 0.001, η*_*p*_*^2^ = 0.448].

N1/P1 was assumed to be related to spatial attention, enhanced by alterations to the spatial factor (Omoto et al., 2001). The clear negative response observed around 180 ms after stimuli onset underlined the validity of our experiments.

### 370–550 ms

In the N400 time window (370–550 ms), the condition × side (rvf-LH presentation, lvf-RH presentation) × region (frontal, central, and parietal) ANOVA revealed a significant main effect of condition [*F*(2,38) = 10.266, *p* = 0.001, η*_*p*_*^2^ = 0.351]. Scientific metaphors elicited more negative N400 (*M* = 0.260, SD = 0.469) than conventional metaphors (*M* = 1.613, SD = 0.557) and literals (*M* = 2.329, SD = 0.701). *Post hoc* analysis showed significant difference between N400s elicited by scientific metaphors and literal pairs (*p* = 0.004) as well as scientific metaphors and conventional metaphors (*p* = 0.010), while no significant difference was found between N400s elicited by conventional metaphors and literal metaphors (*p* = 0.304). There was no significant difference between sides [*F*(1,19) = 0.732, *p* = 0.403, η*_*p*_*^2^ = 0.037] but a marginally significant interaction between condition and side [*F*(2,38) = 2.650, *p* = 0.099, η*_*p*_*^2^ = 0.122]. *Post hoc* analysis showed differences of N400s elicited by scientific metaphors, conventional metaphors and literal pairs were more significant during rvf-LH presentation (*p* = 0.008) than lvf-RH presentation (*p* = 0.015).

Further ANOVA for scientific metaphors and literal pairs revealed no significant condition × side interaction (*p* = 0.353). The differences between N400s elicited by scientific metaphors and literal pairs were significant in both visual field presentations. Moreover, the ANOVA for conventional metaphors and literal pairs showed that conventional metaphors elicited significantly higher N400s than literal pairs during lvf-RH presentation (*p* = 0.050) as opposed to rvf-LH presentation (*p* = 0.581).

**FIGURE 4:**
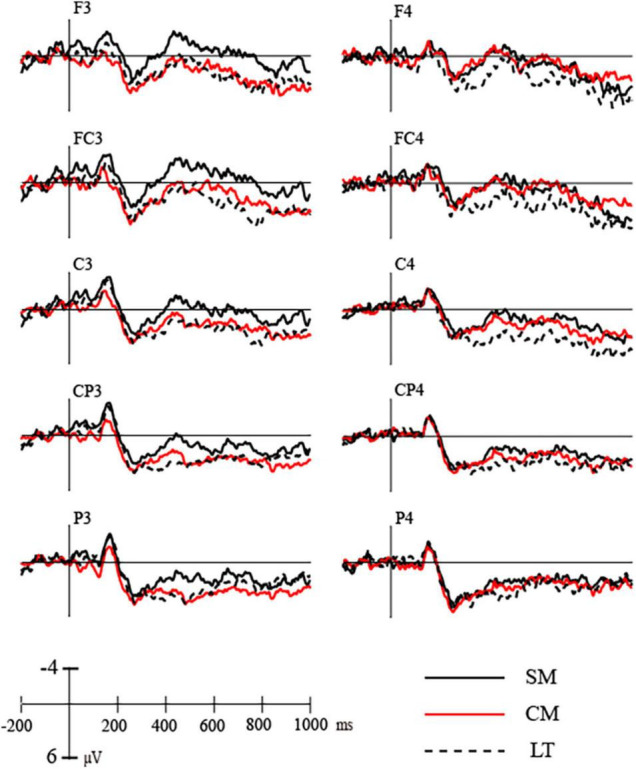
Grand average ERPs elicited by stimuli during left hemisphere presentation (F3–P3) and right hemisphere presentation (F4–P4).

**FIGURE 5:**
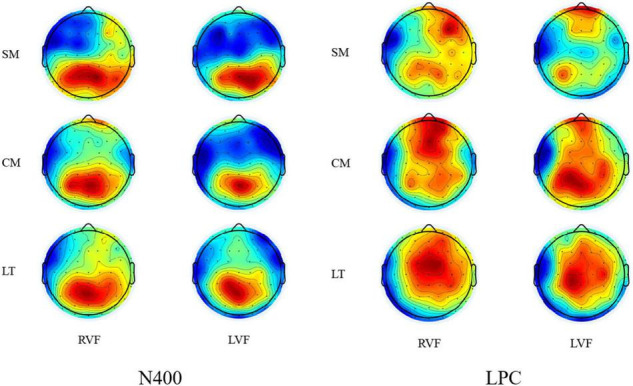
Topographic plots of stimuli for the N400 (370–550 ms) window and the LPC (700–900 ms) window.

More interestingly, the ANOVA for scientific metaphors and conventional metaphors revealed a significant main effect of condition [*F*(1,19) = 11.359, *p* = 0.003, η*_*p*_*^2^ = 0.374] and also a marginally significant interaction between condition and side [*F*(1,19) = 3.369, *p* = 0.082, η*_*p*_*^2^ = 0.151]. *Post hoc* analysis showed no significant difference between N400s elicited by scientific metaphors (*M* = 0.808, SD = 0.490) and conventional metaphors (*M* = 1.454, SD = 0.615) during lvf-RH presentation (*p* = 0.229), but a significant difference between N400s elicited by scientific metaphors (*M* = −0.288, SD = 0.574) and conventional metaphors (*M* = 1.773, SD = 0.768) during rvf-LH presentation (*p* = 0.002) (as shown in Figures 4, 5).

### 700–900 ms

In the LPC time window (700–900 ms), the condition × side × region ANOVA revealed a significant main effect of condition [*F*(2,38) = 5.242, *p* = 0.019, η*_*p*_*^2^ = 0.216] with no interactions. *Post hoc* analysis showed marginally significant difference (*p* = 0.074) between LPCs elicited by scientific metaphors and literal pairs (*M* = 3.095, SD = 0.558) and marginally significant difference (*p* = 0.062) between those by scientific metaphors (*M* = 1.706, SD = 0.429) and conventional metaphors (*M* = 2.878, SD = 0.435) and no significant difference between those by conventional metaphors and literal pairs (*p* = 1.000).

Similar to the results of N400, ANOVA for scientific metaphors and literal pairs revealed a significant main effect of condition [*F*(1,19) = 5.945, *p* = 0.025, η*_*p*_*^2^ = 0.238]. Scientific metaphors elicited a significantly lower LPC than literal pairs during lvf-RH presentation (*p* = 0.017) but not during rvf-LH presentation (*p* = 0.177). For conventional metaphors and literal pairs, there was a marginally significant condition × side interaction [*F*(1,19) = 4.354, *p* = 0.051, η*_*p*_*^2^ = 0.186] with conventional metaphors eliciting distinguished higher LPC only during rvf-LH presentation (*p* = 0.084).

Moreover, the ANOVA for scientific metaphors and conventional metaphors revealed a significant main effect of condition [*F*(1,19) = 6.356, *p* = 0.021, η*_*p*_*^2^ = 0.251]. Scientific metaphors elicited significantly lower LPC than conventional metaphors during rvf-LH presentation (*p* = 0.048) but not during lvf-RH presentation (*p* = 0.145). See Table 4 below.

**TABLE 4:** *Post hoc* analysis results of LPCs elicited by the three conditions.

	LH presentation		RH presentation	
SM	*M* = 1.563, SD = 0.522		*M* = 1.849, SD = 0.471	
		*p* = 0.048		*p* = 0.145
CM	*M* = 2.969, SD = 0.584		*M* = 2.787, SD = 0.497	
		*p* = 0.084		*p* = 0.118
LT	*M* = 2.380, SD = 0.572		*M* = 3.811, SD = 0.741	

## Discussion

The current DVF study introduced scientific metaphors as novel metaphors to examine hemispheric asymmetry. The behavioral results showed increased reaction time and decreased accuracy rate for scientific metaphor comprehension compared to the processing of conventional metaphors and literal pairs, indicating a special mechanism of novel metaphoric processing. But due to limitations of behavioral data, it failed to show any significant differences between rvf-LH and lvf-RH presentation.

The use of ERP in our study clearly revealed the role of each hemisphere in metaphor processing in a temporally dynamic way. Our study reported the same reversing asymmetries of the visual N1 on either hemifield as in Coulson and Van Petten’s (2007) study. In the right hemisphere, stimuli with lvf-RH presentation elicited a larger N100 compared to stimuli with rvf-LH presentation, while in the left hemisphere, stimuli with rvf-LH presentation elicited a larger N100 compared to that with lvf-RH presentation. Although the effect was insensitive to word pair types, it confirmed that lateral processing is evident when stimuli were presented to the opposite visual field, indicating the validity of the study at least in the early stage. In the N400 time window, the results showed that scientific metaphors elicited higher N400 than literal pairs during both rvf-LH and lvf-RH presentation while conventional metaphors elicited higher N400 than literal pairs only during lvf-RH presentation. More interestingly, scientific metaphors elicited higher N400 than conventional metaphors with an interaction between conditions and sides showing a significant difference only during rvf-LH presentation. In the LPC time window, the data suggest that both hemispheres are involved when processing figurative languages, but the right hemisphere takes a more privileged role. The significant activation of the left hemisphere for scientific metaphor processing supports the fine-coarse coding hypothesis (Beeman, 2005). During rvf-LH presentation, the left hemisphere, responsible for the processing of closely related domains, has to integrate the loosely associated domains of scientific metaphor, which greatly increases cognitive taxes.

### N400 (370–550 ms)

Consistent with our prediction, scientific metaphors elicited higher a N400 than conventional metaphors and literal pairs in both hemispheres, indicating a unique mechanism of processing novel metaphors. The result is consistent with some of our previous studies with a central presentation in eliciting larger N400s for both scientific metaphors and conventional metaphors as compared to literal pairs in sentence context (Tang et al., 2017a).

With presentation to rvf-LH, scientific metaphors elicited higher N400 than both conventional metaphors and literal pairs supporting the right hemisphere advantage in novel metaphoric processing in the early stage. According to Aurnhammer et al. (2021), lexical retrieval is indexed by the N400, which is sensitive to linguistic properties like frequency, association, and expectancy. Compared with conventional metaphors and literal pairs, the primary words of scientific metaphors are usually scientific terms such as “*electronics*” and hereby have lower frequency as well as weaker association with base word “*current*” than conventional metaphors or literal pairs. This reveals the unique semantic structure of scientific metaphors that involves two different contexts (Tang et al., 2017a), which may cause greater cognitive taxes for lexical retrieval at the left hemisphere.

During lvf-RH presentation, scientific metaphors and conventional metaphors also elicited higher N400s than literal pairs indicating the unique processing of figurative language in the right hemisphere. According to *the structure-mapping theory* (Wolff and Gentner, 2011), processing a metaphor involves an initial alignment between target and base domains. Therefore, the processing of literal pairs may only involve information retrieval and mainly activate the left hemisphere, while the processing of metaphor may involve not only information retrieval but also the structure alignment, which may activate the right hemisphere to compare the target and base to find correspondences at this stage.

In addition, conventional metaphors elicited higher N400 than literal pairs only in the right hemisphere. This finding proves that conventional metaphors have a different processing mechanism from literal meaning processing: the right hemisphere is involved in semantic integration. And more importantly, there was no significant difference between N400s elicited by scientific metaphors and conventional metaphors during lvf-RH presentation, but a significant difference between N400s elicited by scientific metaphors and conventional metaphors during rvf-LH presentation, which reveals a special role of the right hemisphere, especially in novel metaphor comprehension. This is to say, compared to the left hemisphere, the right hemisphere is more sensitive to the semantic integration between conceptual domains over a long distance.

Our findings, however, are not aligned with Coulson and Van Petten’s (2007) study in which ERP metaphoricity effects were very similar across hemispheres despite the left-hemisphere advantage in processing low-cloze literals. One possible reason for the contradictory results lies in the different stimuli used in the two studies. In their research, the concepts of both source domain and target domain of the low-cloze metaphors were mostly from everyday contexts. Firstly, the metaphoricity of those novel metaphors might not have been high enough to elicit significantly different ERP components. Scientific metaphors are of different mapping distances and contextual complexity, which can amplify processing differences between metaphors and literal meaning. Secondly, the individual differences of the participants in terms of their general knowledge might also have brought some impact on the experimental results. For example, a bibliophile might have a totally different processing mechanic from a bibliophobe when processing poetic metaphors. Those individual differences might not be able to be manifested through education or age, and ignorance of these types of differentials might lead to inaccuracy. However, in our study, the scientific metaphors we used as the experimental corpus were obtained from common scientific concepts which are taught in middle school or high school in China. More importantly, there is a learning process before the formal experiment, which can effectively reduce the processing differences caused by different general contextual knowledge of the participants. Besides, the sentence context used in the experiments might be another reason why no significant differences in N400 elicited by low-cloze metaphors compared to literal meaning in the study. The left hemisphere advantage has been found in integrating sentence contexts (Forgács et al., 2014), which might eliminate the differences caused by the conditions.

### Late Positive Component (700–900 ms)

Unlike in previous DVF experiments, our study also reported distinguished differences in late ERP component, which were also reported in some of our previous studies using central presentation in eliciting a smaller LPC for both scientific metaphors and conventional metaphors as compared to literal pairs (Tang et al., 2017a). A possible reason is that late negativity overlapped in the window of LPC indicating the scientific inference for knowledge understanding.

In the LPC time window, the data revealed different hemispheric activation between scientific metaphors and conventional metaphors. Compared with literal pairs, conventional metaphors elicited significantly higher LPCs during the rvf-LH presentation, while the scientific metaphors elicited significantly lower LPCs during the lvf-RH presentation, which indicates the right hemisphere advantage in the late stage of metaphoric processing. According to the structure-mapping theory (Wolff and Gentner, 2011), the processing of metaphors involves a later directional mapping from base to target. *The career of metaphor* (Bowdle and Gentner, 2005; Wolff and Gentner, 2011) added that the bases of highly conventional metaphors already possess a salient conventional metaphoric meaning, whereas the metaphoric abstraction must be derived anew for a novel figurative. Hence a novel metaphor is understood with the analogy, while a conventional metaphor is understood with a category statement (Bowdle and Gentner, 1999, 2005), which is computationally less demanding and even easier than “automatic” (Holyoak and Stamenkovic, 2018). Combined with the fine-coarse coding hypothesis (Beeman, 2005), the mapping of conventional metaphors is simpler than the processing of literal expressions and therefore may mainly activate the left hemisphere. In contrast, the mapping of scientific metaphors may involve a comparison process to establish a new integration which is more computationally costly than literal expressions and therefore may mainly activate the right hemisphere. More importantly, scientific metaphors elicited markedly lower LPCs than conventional metaphors during the rvf-LH presentation, showing greater cognitive taxes for the left hemisphere to process analogy mapping of scientific metaphors.

In summary, the hemispheric processing of scientific metaphors might differ from that of conventional metaphors and literal expressions in the following three ways. First, the information retrieval for scientific terms involved in scientific metaphors might increase the cognitive loads of the left hemisphere. Secondly, during the processing of semantic integration and structure alignment, the distance between scientific target domains and daily source domains of a scientific metaphor might be longer than that between the two daily domains of a conventional metaphor, which might result in a higher calculative tax for the right hemisphere. Thirdly, during the later-stage processing of directional mapping, the mapping of scientific metaphors may involve a comparison process to establish a new integration which is assumed to be computationally costly for the right hemisphere. In general, the processing of scientific metaphors might activate both hemispheres and the right hemisphere plays an important role in semantic integration, structure alignment and directional mapping.

## Conclusion

The current DVF study introduced scientific metaphors as novel metaphors presenting orientation mapping from the specific and familiar domains to the abstract and unfamiliar domains to examine hemispheric asymmetry. In addition, a scientific metaphor has a more complex context which increases the cognitive load, especially in the late stage. The results suggest that the complexity of mapping impacts the lateralization of metaphor processing. Although both hemispheres are involved in scientific metaphor comprehension, the right hemisphere takes a special role in integrating domains across a long distance and making inferences for scientific knowledge.

However, the DVF paradigm used in the study has some limitations. When a stimulus is presented on one visual field, the opposite side of the hemisphere to the visual field is activated while the same side of the hemisphere is inhibited, which might result in some counteraction of effects. This might also explain why similar studies draw conclusions that both hemifields are involved in non-literal language processing. Therefore, the extent to which the hemispheres are inhibited by hemifield presentation should be taken into data analysis is worthy of further verification. Besides, it is difficult to ensure that the stimuli presented in different visual fields would be processed by the opposite hemispheres totally due to individual physical and attentional differences. Some participants might sometimes roll their eyes unconsciously. Also, due to limited time, our study did not modulate SOA as some previous DVF experiments did. Future studies are suggested to apply different SOAs to further study the time course of non-figurative language processing, as well as to use more advanced techniques such as eye-tracking to monitor the whole experimental process.

## Data Availability Statement

The raw data supporting the conclusions of this article will be made available by the authors, without undue reservation.

## Ethics Statement

The studies involving human participants were reviewed and approved by the Ethics Committee of Shaanxi Normal University. The patients/participants provided their written informed consent to participate in this study.

## Author Contributions

XT, MH, SX, and SH contributed to conception and design of the study. XT, MH, and SX performed the data collection. LS, MH, and YH performed the analysis. MH and LS wrote the first draft of the manuscript. All authors contributed to manuscript revision, read, and approved the submitted version.

## Conflict of Interest

The authors declare that the research was conducted in the absence of any commercial or financial relationships that could be construed as a potential conflict of interest.

## Publisher’s Note

All claims expressed in this article are solely those of the authors and do not necessarily represent those of their affiliated organizations, or those of the publisher, the editors and the reviewers. Any product that may be evaluated in this article, or claim that may be made by its manufacturer, is not guaranteed or endorsed by the publisher.

## References

[B1] Anaki D. Faust M. Kravetz S. (1998). Cerebral hemispheric asymmetries in processing lexical metaphors. *Neuropsychologia* 36 353–362. 10.1016/S0028-3932(97)00110-3 9665646

[B2] Aurnhammer C. Delogu F. Schulz M. Brouwer H. Crocker M. W. (2021). Retrieval (N400) and integration (P600) in expectation-based comprehension. *PLoS One* 16:e0257430. 10.1371/journal.pone.0257430 34582472 PMC8478172

[B3] Beeman M. J. (1998). “Coarse semantic coding and discourse comprehension,” in *Right Hemisphere Language Comprehension: Perspectives From Cognitive Neuroscience*, eds Beeman M. J. Chiarello C. (Mahwah: Lawrence Erlbaum Associates Publishers), 255–284.

[B4] Beeman M. J. (2005). Bilateral brain processes for comprehending natural language. *Trends Cogn. Sci.* 9 512–518. 10.1016/j.tics.2005.09.009 16214387

[B5] Bohrn I. C. Altmann U. Jacobs A. M. (2012). Looking at the brains behind figurative language—A quantitative meta-analysis of neuroimaging studies on metaphor, idiom, and irony processing. *Neuropsychologia* 50 2669–2683. 10.1016/j.neuropsychologia.2012.07.021 22824234

[B6] Bowdle B. F. Gentner D. (1999). “Metaphor comprehension: From comparison to categorization,” in *Proceedings of the Twenty-First Annual Conference of the Cognitive Science Society*, eds Hahn M. Stoness S. C. (Hillsdale: Lawrence Erlbaum Associates), 90–95. 10.4324/9781410603494-21

[B7] Bowdle B. F. Gentner D. (2005). The Career of Metaphor. *Psychol. Rev.* 112 193–216. 10.1037/0033-295X.112.1.193 15631593

[B8] Coulson S. Federmeier K. D. Van Petten C. Kutas M. (2005). Right Hemisphere Sensitivity to Word- and Sentence-Level Context: evidence From Event-Related Brain Potentials. *J. Exp. Psychol. Learn. Mem. Cogn.* 31 129–147. 10.1037/0278-7393.31.1.129 15641911

[B9] Coulson S. Van Petten C. (2007). A special role for the right hemisphere in metaphor comprehension? ERP evidence from hemifield presentation. *Brain Res.* 1146 128–145. 10.1016/j.brainres.2007.03.008 17433892

[B10] Eviatar Z. Just M. A. (2006). Brain correlates of discourse processing: an fMRI investigation of irony and conventional metaphor comprehension. *Neuropsychologia* 44 2348–2359. 10.1016/j.neuropsychologia.2006.05.007 16806316 PMC1894906

[B11] Faust M. Mashal N. (2007). The role of the right cerebral hemisphere in processing novel metaphoric expressions taken from poetry: a divided visual field study. *Neuropsychologia* 45 860–870. 10.1016/j.neuropsychologia.2006.08.010 17010392

[B12] Forgács B. Lukács A. Pléh C. (2014). Lateralized processing of novel metaphors: disentangling figurativeness and novelty. *Neuropsychologia* 56 101–109. 10.1016/j.neuropsychologia.2014.01.003 24418155

[B13] Giora R. (1997). Understanding figurative and literal language: the graded salience hypothesis. *Cogn. Linguist.* 8 183–206. 10.1515/cogl.1997.8.3.183 31158291

[B14] Giora R. (2003). *On Our Mind: Salience, Context, and Figurative Language.* New York: Oxford University Press.

[B15] Holyoak K. J. Stamenkovic D. (2018). Metaphor comprehension: a critical review of theories and evidence. *Psychol. Bull.* 144 641–671. 10.1037/bul0000145 29517263

[B16] Ianni G. R. Cardillo E. R. McQuire M. Chatterjee A. (2014). Flying under the radar: figurative language impairments in focal lesion patients. *Front. Hum. Neurosci.* 8:871. 10.3389/fnhum.2014.00871 25404906 PMC4217389

[B17] Kazmerski V. A. Blasko D. G. Dessalegn B. G. (2003). ERP and behavioral evidence of individual differences in metaphor comprehension. *Mem. Cogn.* 31 673–689. 10.3758/BF03196107 12956233

[B18] Lai V. T. Curran T. (2013). ERP evidence for conceptual mappings and comparison processes during the comprehension of conventional and novel metaphors. *Brain Lang.* 127 484–496. 10.1016/j.bandl.2013.09.010 24182839

[B19] Lai V. T. Van Dam W. Conant L. L. Binder J. R. Desai R. H. (2015). Familiarity differentially affects right hemisphere contributions to processing metaphors and literals. *Front. Hum. Neurosci.* 9:44. 10.3389/fnhum.2015.00044 25713522 PMC4322727

[B20] Lauro L. J. R. Tettamanti M. Cappa S. F. Papagno C. (2008). Idiom Comprehension: a Prefrontal Task? *Cereb. Cortex* 18 162–170. 10.1093/cercor/bhm042 17490991

[B21] Mashal N. Faust M. (2008). Right hemisphere sensitivity to novel metaphoric relations: application of the signal detection theory. *Brain Lang.* 104 103–112. 10.1016/j.bandl.2007.02.005 17445878

[B22] Mitchell R. Vidaki K. Lavidor M. (2016). The Role of Left and Right Dorsolateral Prefrontal Cortex in Semantic Processing: a transcranial direct current stimulation study. *Neuropsychologia* 91 480–489. 10.1016/j.neuropsychologia.2016.08.019 27553267

[B23] Oldfield R. C. (1971). The assessment and analysis of handedness: the Edinburgh inventory. *Neuropsychologia* 9 97–113. 10.1016/0028-3932(71)90067-4 5146491

[B24] Omoto S. Kuroiwa Y. Li M. Doi H. Shimamura M. Koyano S. (2001). Modulation of event-related potentials in normal human subjects by visual divided attention to spatial and color factors. *Neurosci. Lett.* 311 198–202. 10.1016/S0304-3940(01)02172-3 11578828

[B25] Rapp A. M. Leube D. T. Erb M. Grodd W. Kircher T. T. (2004). Neural correlates of metaphor processing. *Cogn. Brain Res.* 20 395–402. 10.1016/j.cogbrainres.2004.03.017 15268917

[B26] Rutter B. Kröger S. Hill H. Windmann S. Hermann C. Abraham A. (2012). Can clouds dance? Part 2: an ERP investigation of passive conceptual expansion. *Brain Cogn.* 80 301–310. 10.1016/j.bandc.2012.08.003 23137771

[B27] Schneider S. Rapp A. M. Haeußinger F. B. Ernst L. H. Hamm F. Fallgatter A. J. (2014). Beyond the N400: complementary access to early neural correlates of novel metaphor comprehension using combined electrophysiological and haemodynamic measurements. *Cortex* 53 45–59. 10.1016/j.cortex.2014.01.008 24566043

[B28] Sela T. Panzer M. S. Lavidor M. (2017). Divergent and convergent hemispheric processes in idiom comprehension: the role of idioms predictability. *J. Neurolinguistics* 44 134–146. 10.1016/j.jneuroling.2017.05.002

[B29] Tang X. Qi S. Jia X. Wang B. Ren W. (2017a). Comprehension of scientific metaphors: complementary processes revealed by ERP. *J. Neurolinguistics* 42 12–22. 10.1016/j.jneuroling.2016.11.003

[B30] Tang X. Qi S. Wang B. Jia X. Ren W. (2017b). The temporal dynamics underlying the comprehension of scientific metaphors and poetic metaphors. *Brain Res.* 1655 33–40. 10.1016/j.brainres.2016.11.005 27845031

[B31] Wolff P. Gentner D. (2011). Structure-mapping in metaphor comprehension. *Cogn. Sci.* 35 1456–1488. 10.1111/j.1551-6709.2011.01194.x 21929665

[B32] Yang J. (2014). The role of the right hemisphere in metaphor comprehension: a meta-analysis of functional magnetic resonance imaging studies. *Hum. Brain Mapp.* 35 107–122. 10.1002/hbm.22160 22936560 PMC6868953

[B33] Yang J. Li P. Fang X. Shu H. Liu Y. Chen L. (2016). Hemispheric involvement in the processing of Chinese idioms: an fMRI study. *Neuropsychologia* 87 12–24. 10.1016/j.neuropsychologia.2016.04.029 27143223

